# Presenting symptoms as prognostic measures of mental health recovery among service members with concussion

**DOI:** 10.3389/fneur.2022.1070676

**Published:** 2023-01-13

**Authors:** Rosemay A. Remigio-Baker, Lars D. Hungerford, Mark L. Ettenhofer, Lori L. Barnard, Ida Babakhanyan, Brian Ivins, Keith Stuessi, Carlos Diego J. Monasterio, Jason M. Bailie

**Affiliations:** ^1^Traumatic Brain Injury Center of Excellence (TBICoE), Silver Spring, MD, United States; ^2^General Dynamics Information Technology, Falls Church, VA, United States; ^3^Naval Medical Center at San Diego, San Diego, CA, United States; ^4^University of California, San Diego, La Jolla, CA, United States; ^5^Naval Hospital Camp Pendleton, Intrepid Spirit 7, Camp Pendleton, CA, United States

**Keywords:** traumatic brain injury, concussion, post-traumatic stress disorder (PSTD), depressive symptoms, military, service members

## Abstract

**Background:**

Comorbid mental illness may negatively impact recovery from concussion. This study evaluated whether the level of symptom clusters at clinic intake contribute to poor mental health recovery in concussed patients during treatment, which may in turn serve as a target intervention.

**Objective:**

The objective of this study is to examine the association between the level of initial symptoms and mental health symptoms among service members with concussion.

**Methods:**

Data were obtained from 483 active duty service members treated in interdisciplinary treatment programs for traumatic brain injury, all of which were concussions. Pre-treatment symptom clusters included self-reported hyperarousal, dissociation/depression, cognitive dysfunction/headache and neurological symptoms. The outcomes, clinically-relevant decreases in depressive symptoms (assessed by the 8-item Patient Health Questionnaire, PHQ-8) and PTSD symptoms (assessed by the PTSD Checklist for DSM-5, PCL-5), were defined as a decrease in PHQ-8 > 5 and PCL-5 > 7, respectively. Poisson regression with robust error variance was used to evaluate the relationship between the level of each symptom cluster and clinically-relevant decrease in outcomes.

**Results:**

Participants with higher (vs. lower) levels of pre-treatment hyperarousal and dissociation/depression symptom cluster were less likely to improve in depressive and PTSD symptoms during treatment. The level of cognitive/headache and neurological symptom clusters were not significantly associated with any symptom changes.

**Conclusion:**

These findings support the need for individualized treatment for symptoms identified and treated after determining concussion history, with particular attention to high levels of hyperarousal and dissociation/depression prior to treatment.

## 1. Introduction

Mental health conditions such as depression and post-traumatic stress disorder (PTSD) can have a profound impact on overall individual wellbeing, which may hinder recovery from other illnesses (1–7). As such, individuals who suffer from such ailments have an increased risk for premature mortality (3, 8). Depression and PTSD, in particular, may be a part of the neurologic or psychiatric sequelae of traumatic brain injury (TBI), which may partly explain the concurrent nature of these psychiatric symptoms and post-concussion symptoms (9–12). Neuropsychiatric findings have shown a potential biological link between TBI and mood disorders such as depression (13). This includes a reduction of the left prefrontal gray matter volume among survivors of TBI, of any severity, with depression (14), as well as observing frequent diffuse axonal injury and damage in the frontal and anterior temporal regions in this population (10). Abnormalities in brain areas such as relatively “smaller hippocampal and anterior cingulate volumes, increased amygdala function, and decreased medial prefrontal/anterior cingulate function” have been implicated for PTSD (15). Disturbances in neurotransmission systems (10), along with disruption in hippocampal functioning and morphology (15, 16) are also proposed biological mechanisms that contribute to increased depressive disorders and PTSD among individuals who sustain a TBI. Depression and PTSD may also exist prior to a TBI event; thus, deterioration of mental health symptoms post-TBI may be exacerbated by preexisting mental health conditions. In a surveillance study at a hospital level I trauma center and a specialized rehabilitation unit, patients with TBI who had major depression during the first year post-injury were more likely to have a history of mood and anxiety disorder compared to those without major depression (14). This finding was supported by another study showing major depressive disorder diagnosed during the first year after TBI to be associated with having a history of major depressive disorder among hospitalized adults with complicated mild to severe TBI (17). Whether mental health symptoms are pre-existing or borne from TBI, they may lead to difficulties in recovering from symptoms identified and treated after determining the history of TBI (1–7). The level of mental health symptoms at clinical presentation for TBI, whether as a proxy for an underlying health issue or as a sequalae of TBI, may provide a marker to which to identify individuals who may be at high risk for poorer mental health outcomes during TBI treatment, and who may need additional resources outside of TBI clinics to fully address such issues either prior to or concurrently with TBI treatment.

The occupational demands and hazards associated with military service increase the risk for both TBI and mood disorders among active duty service members and veterans. If not treated appropriately, mental illness may impede full recovery from TBI symptoms (18). Long-term follow-up of active duty service members and veterans with a history of TBI shows that mental health factors (primarily PTSD) remain one of the strongest predictors of poor recovery as much as 15 years after injury (19). The military healthcare system includes interdisciplinary treatment programs for TBI that provide a multifaceted treatment approach (20). This may include direct mental health treatment, many adjunctive treatments such as physical and music therapy, and acupuncture which may improve mood complaints (21–23). As TBI significantly impacts warfighter readiness, these interdisciplinary clinics, specializing in the treatment of TBI, have become a central component in the military's TBI pathway of care (20).

Mild TBI, or concussion, is the most prevalent form of TBI (24). Concussive symptoms at clinic intake vary by individual, as patients may present with varied symptoms including cognitive, emotional, and/or somatic complaints (25). If predictors can be identified to inform prognosis and symptom trajectory based on this initial presentation, it may be possible to improve outcomes through individualized care. However, it is unclear if different symptom elevations at admission are predictive of treatment response for mental health recovery. It is imperative that characteristics that define vulnerable groups prone to poor outcome are identified and treated early for predictive variables such as high levels of specific symptoms (e.g., hyperarousal, dissociation/depression, cognitive dysfunction/headache, and neurological) prior to treatment to optimize recovery from a concussion, and expedite return to duty or activity.

The objective of this study was to determine, in an outpatient population receiving care for concussion, whether the levels of specific symptom clusters prior to treatment can serve as a prognostic measure of changes in mental health symptoms during treatment. We hypothesized that high (vs. low) levels of pre-treatment symptom clusters (i.e., hyperarousal, dissociation/depressive, cognitive dysfunction/headache, and neurological) would be associated with reduced benefit from treatment (i.e., less symptom reduction) in mental health outcome such as depressive and PTSD symptoms across the course of treatment.

## 2. Materials and methods

### 2.1. Participants

Study participants were patients from interdisciplinary TBI programs at two large military treatment outpatient facilities located in the Southwestern US. The available rehabilitative treatments from these sites included occupational, physical, speech, cognitive and music therapy, acupuncture and behavioral therapy to name a few. Data were obtained from an Institutional Review Board-approved clinical registry study of patients who received care between January 2017 and January 2020 (*n* = 603) where, upon enrollment at the facility, patients agreed to have their data used for research. Patients who agreed to participate were provided with the Project Information Sheet and providers were available to answer any questions. A waiver of informed consent applied for this study.

**Figure 1:**
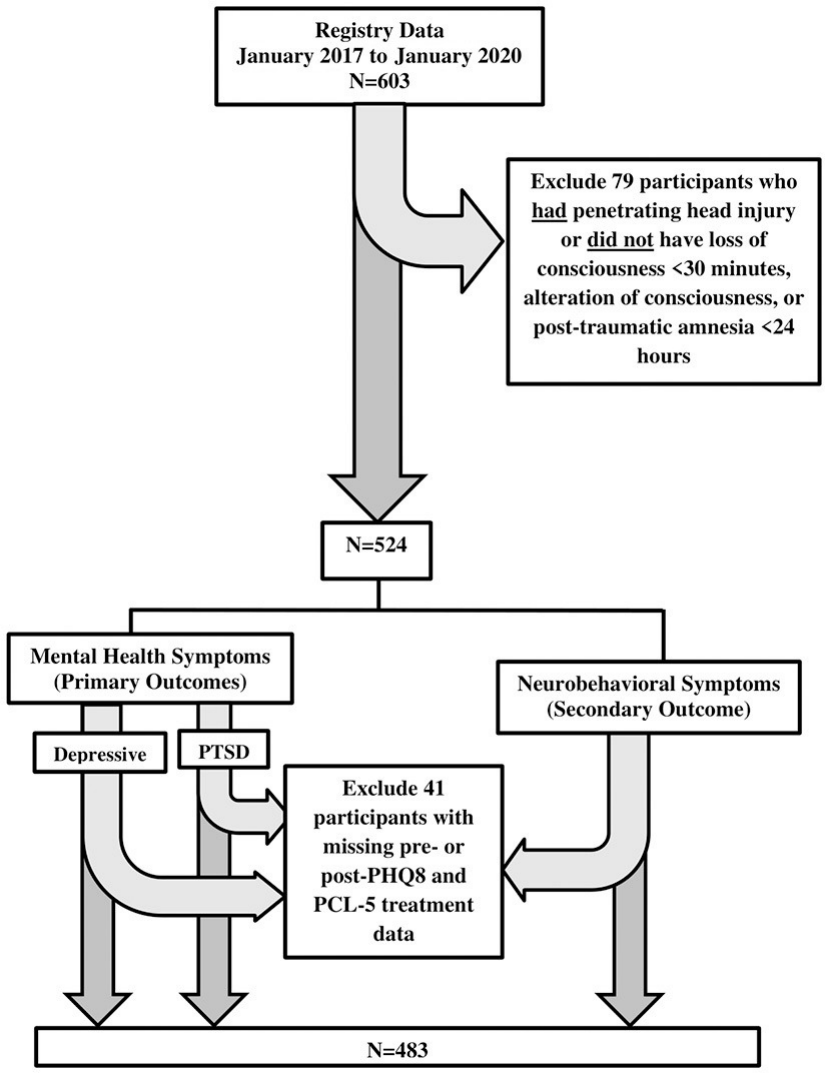
Study sample size flowchart. PHQ-8, patient health questionnaire (8 items); PCL-5, post-traumatic stress disorder checklist, DSM-V; NSI, neurobehavioral symptom inventory.

Eligible participants had a non-penetrating head injury and with loss of consciousness (< 30 min), alteration of consciousness (i.e., dazed), or post-traumatic amnesia (< 24 h) (*n* = 524). Diagnosis of concussion, which included assessment of loss or alteration of consciousness or post-traumatic amnesia, was determined using the Ohio State University TBI Identification Method structured interview shown to have high reliability and validity to assess lifetime concussion history (26). An additional 41 patients were excluded from analyses due to missing depressive and PTSD pre- and post-treatment measures (missing depressive symptom measures: *n* = 33 pre-treatment and *n* = 3 post-treatment; missing PTSD symptom measures: *n* = 12 pre-treatment and *n* = 3 post-treatment; missing neurobehavioral symptom measures: *n* = 13 pre-treatment and *n* = 3 post-treatment), leaving a total sample of 483 observations for analyses. There were no statistically significant differences in sample characteristics between those with either only pre- or post-treatment outcome measures and those with both pre- and post-treatment outcome measures, with the exception of Neurobehavioral Symptom Inventory (NSI) where having only pre-treatment measure vs. having both pre- and post-treatment measures was more frequent in one site over the other. Adjustments were made when site was significantly related to the level of symptom cluster. Figure 1 provides a flow chart of how the data sample was determined.

### 2.2. Measures

#### 2.2.1. Independent variable: Level of symptom clusters prior to treatment

This study evaluated the level of self-reported symptoms *prior to treatment* categorized based on groups pre-determined using cluster analyses by Bailie et al. (25). Symptom clusters were adopted to capture the full spectrum of symptoms commonly experienced by service members seeking treatment for a TBI. Briefly, four symptom clusters were identified using items from the 22-item Neurobehavioral Symptom Inventory (NSI) and the 17-item Posttraumatic Stress Disorder Checklist-Civilian Version (PCL-C): the hyperarousal symptom cluster (e.g., disturbing dreams/memories of stressful events, feeling stressful events were happening again, and feeling jumpy or easily startled), the depression/dissociation symptom cluster (e.g., feeling distant from others, feeling emotionally numb, and loss of interest in things used to enjoy), the cognitive/headache symptom cluster (e.g., forgetfulness, headaches, poor concentration/easily distracted), and the neurological symptom cluster (e.g., change in taste and/or smell, numbness or tingling in parts of the body, and loss of balance) (25). This study utilized 17 corresponding items from the PCL-C that are included in the PTSD Checklist, DSM-5 (PCL-5) which was administered in this study. Supplementary Table 1 shows each survey item of NSI and PCL-5 (corresponding to PCL-C) under each symptom cluster, as adapted from Bailie et al. (25). The level for each symptom cluster was converted to a *z*-score (using the total mean of each symptom cluster) to make the level of impact of each cluster category comparable. A *z*-score cut-off was used to distinguish between high (*z*-score ≥ 1.5) vs. low (*z*-score < 1.5) levels of pre-treatment symptom clusters. This cut-off took into account the sample size needs (i.e., ≥5 in each cell) when main variables were analyzed as categories.

#### 2.2.2. Dependent variables (outcome): Mental health and neurobehavioral symptoms

Our two main mental health outcomes were self-reported depressive and PTSD symptoms. The 8-item Patient Health Questionnaire (PHQ-8) was utilized to evaluate depressive symptoms over the past 2 weeks from assessment (27–30). Responses reported the extent to which each symptom bothered the participant, ranging from “not at all” (coded as 0) to “nearly every day” (coded as 3). PHQ-8 has previously demonstrated good internal consistency (Cronbach's alpha of 0.82) and convergent validity with a well-established instrument to assess depressive symptoms (i.e., the Center for Epidemiology Study—Depression survey) (30). A clinically-relevant decrease in depressive symptoms was defined as a PHQ-8 score reduction of at least 5 points (31). The 20-item PCL-5 was used to evaluate PTSD symptoms (32). This survey assessed the extent with which patients were bothered by their symptoms in the past month. The responses ranged from “not at all” (coded as 0) to “extremely” (coded as 4). PCL-5 has previously demonstrated good test-retest reliability (*r* = 0.66–0.96), internal consistency (Cronbach's alpha = 0.83–0.98), discriminant validity when compared with measures of related constructs (< *r* = 0.87), and convergent validity (*r* with other PTSD measures = 0.62–0.93) (33). A clinically-relevant decrease in PTSD symptoms was defined as a PCL-5 score reduction of at least 7 points (34). As a secondary outcome, self-reported neurobehavioral symptoms in the past 2 weeks were evaluated using the NSI. Responses included “none” (coded as 0), “mild,” “moderate,” “severe,” and “very severe” (coded as 4) (35, 36). The NSI has previously demonstrated good internal consistency (total alpha = 0.95; subscale alpha = 0.88–0.92), good validity and test-retest reliability (*r* = 0.78–0.94), and moderate external validity (*r* = 0.41) (37). A clinically-relevant decrease in neurobehavioral symptoms was defined as having at least an 8-point reduction in NSI score (34). Comparable clinically-relevant increases in outcome were infrequent and were therefore not included in analyses.

#### 2.2.3. Covariates

The covariates evaluated in this study included self-reported demographic (i.e., age, gender, race, highest education attained, marital status, and primary language) and military (i.e., rank, branch of service, history of deployment and number of years in active duty) characteristics determined prior to treatment. The race/ethnic groups reported included Non-Hispanic White, Non-Hispanic Black, Hispanic/Latino, Asian, Native Hawaiian/Pacific Islander, and American Indian/Alaska Native. Additionally, the time from injury to intake, length of time in the TBI treatment program, and pre-treatment scores for depressive, PTSD and neurobehavioral symptoms were assessed. The highest level of education was coded as high school equivalency diploma (GED), high school diploma, some college, associate's degree, bachelor's degree, and master's degree or doctoral degree. History of combat deployment was coded as a binary variable (categorized as none or at least one).

### 2.3. Statistical analyses

Chi-square tests and *t*-tests were used to evaluate the relationships between the level of each symptom cluster and categorical and continuous covariates, respectively, to assess potential confounding factors. Analysis of variance (ANOVA) was utilized to test for the association between the level of each symptom cluster and continuous covariates with non-normal distribution. Two separate set of analyses were conducted, one using the entire sample (*n* = 483), and another excluding those with a clinically-relevant increase in symptoms (*n* = 439). Covariates significantly related to the level of symptom clusters were included in appropriate models (see Table 1). ANOVA was also used to assess the mean unit of change in symptoms (i.e., depressive, PTSD and neurobehavioral) per unit change in *z*-score of symptom clusters. As the prevalence of our outcomes was relatively high, Poisson regression with robust error variance was utilized to evaluate the prevalence of a clinically-relevant decrease in symptoms (vs. no change) from pre- to post-treatment (defined as either the data at discharge or at the last follow-up) by the level of each symptom cluster (high vs. low) prior to treatment. Sensitivity analyses were also conducted evaluating data using the entire sample (i.e., those with [*n* = 95] and without [*n* = 388] discharge data) and comparing to results using data of those with only discharge data. This provided a gauge as to any existing difference between those with vs. without discharge data and determine the level of impact on the results. Significance was based on a *p*-value of < 0.05 using two-tailed tests, and all analyses were conducted using Stata statistical software, release v.15 (StataCorp, 2017, College Station, TX).

**Table 1:** Sample characteristics, overall and by initial symptom clusters (*n* = 483).

**Characteristics**	**Estimate**	**Hyperarousal**		**Dissociation/depression**		**Cognitive/headache**		**Neurological**	
	**All (*n* = 483)**	**Low^a^ (*n* = 440)**	**High^a^ (*n* = 43)**	**Low^a^ (*n* = 436)**	**High^a^ (*n* = 47)**	**Low^a^ (*n* = 446)**	**High^a^ (*n* = 37)**	**Low^a^ (*n* = 444)**	**High^a^ (*n* = 39)**
**Study site**, ***n*** **(%)**					
Camp Pendleton	257 (53.2)	230 (52.3)	27 (62.8)	227 (52.1)	30 (63.8)	233 (52.2)	24 (64.9)	236 (53.2)	21 (53.9)
NMCSD	226 (46.8)	210 (47.7)	16 (37.2)	209 (47.9)	17 (36.2)	213 (47.8)	13 (35.1)	208 (46.9)	18 (46.2)
**Age (in years)**					
Mean (SD)	32.2 (8.6)	32.0 (8.7)	34.0 (8.3)	32.0 (8.8)	33.7 (7.5)	32.0 (8.7)	33.9 (8.3)	32.1 (8.6)	33.1 (9.3)
**Gender**, ***n*** **(%)**					
Male	427 (88.4)	390 (88.6)	37 (86.1)	384 (88.1)	43 (91.5)	392 (87.9)	35 (94.6)	393 (88.5)	34 (87.2)
Female	56 (11.6)	50 (11.4)	6 (14.0)	52 (11.9)	4 (8.5)	54 (12.1)	2 (5.4)	51 (11.5)	5 (12.8)
**Race**, ***n*** **(%)**					
Non-Hispanic White	317 (65.6)	**301 (68.4)**	**16 (37.2)** ^**b**^	**294 (67.4)**	**23 (48.9)** ^**b**^	294 (65.9)	23 (62.2)	**301 (67.8)**	**16 (41.0)** ^**b**^
Non-Hispanic Black	50 (10.4)	**41 (9.3)**	**9 (20.9)**	**43 (9.9)**	**7 (14.9)**	46 (10.3)	4 (10.8)	**43 (9.7)**	**7 (18.0)**
Hispanic/Latino	75 (15.5)	**63 (14.3)**	**12 (27.9)**	**65 (14.9)**	**10 (21.3)**	69 (15.5)	6 (16.2)	**67 (15.1)**	**8 (20.5)**
Asian	22 (4.6)	**17 (3.9)**	**5 (11.6)**	**17 (3.9)**	**5 (10.6)**	19 (4.3)	3 (8.1)	**15 (3.4)**	**7 (18.0)**
Native Hawaiian/Pacific Islander	12 (2.5)	**12 (2.7)**	**0 (0)**	**12 (2.8)**	**0 (0)**	12 (2.7)	0 (0)	**12 (2.7)**	**0 (0)**
American Indian/Alaska Native	7 (1.5)	**6 (1.4)**	**1 (2.3)**	**5 (1.2)**	**2 (4.3)**	6 (1.4)	1 (2.7)	**6 (1.4)**	**1 (2.6)**
**Marital status**, ***n*** **(%)**					
Single	127 (26.3)	116 (26.4)	11 (25.6)	119 (27.3)	8 (17.0)	117 (26.2)	10 (27.0)	116 (26.1)	11 (28.2)
Married	327 (67.7)	297 (67.5)	30 (69.8)	289 (66.3)	38 (80.9)	302 (67.7)	25 (67.6)	300 (67.6)	27 (69.2)
Divorced/separated	29 (6.0)	27 (6.1)	2 (4.7)	28 (6.4)	1 (2.1)	27 (6.1)	2 (5.4)	28 (6.3)	1 (2.6)
**Education**, ***n*** **(%)**					
GED	2 (0.4)	1 (0.2)	1 (2.3)	1 (0.2)	1 (2.1)	1 (0.2)	1 (2.7)	1 (0.2)	1 (2.6)
High school diploma	161 (33.3)	141 (32.1)	20 (46.5)	146 (33.5)	15 (31.9)	148 (33.2)	13 (35.1)	144 (32.4)	17 (43.6)
Some college	12 (33.5)	148 (33.6)	14 (32.6)	148 (33.9)	14 (29.8)	147 (33.0)	15 (40.5)	149 (33.6)	13 (33.3)
Associate's degree	36 (7.5)	35 (8.0)	1 (2.3)	32 (7.3)	4 (8.5)	34 (7.6)	2 (5.4)	34 (7.7)	2 (5.1)
Bachelor's degree	87 (18.0)	82 (18.6)	5 (11.6)	79 (18.1)	8 (17.0)	83 (18.6)	4 (10.8)	83 (18.7)	4 (10.3)
Master's degree	29 (6.0)	27 (6.1)	2 (4.7)	24 (5.5)	5 (10.6)	27 (6.1)	2 (5.4)	27 (6.1)	2 (5.1)
Doctoral degree	6 (1.2)	6 (1.4)	0 (0)	6 (1.4)	0 (0)	6 (1.4)	0 (0)	6 (1.4)	0 (0)
**Branch of service**, ***n*** **(%)**					
Navy	225 (46.6)	206 (46.8)	19 (44.2)	204 (46.8)	21 (44.7)	210 (47.1)	15 (40.5)	205 (46.2)	20 (51.3)
Marines	236 (48.9)	214 (48.6)	22 (51.2)	212 (48.6)	24 (51.1)	215 (48.2)	21 (56.8)	219 (49.3)	17 (43.6)
Army	14 (2.9)	12 (2.7)	2 (4.7)	12 (2.8)	2 (4.3)	13 (2.9)	1 (2.7)	12 (2.7)	2 (5.1)
Air force	7 (1.5)	7 (1.6)	0 (0)	7 (1.6)	0 (0)	7 (1.6)	0 (0)	7 (1.6)	0 (0)
Coast guard	1 (0.2)	1 (0.2)	0 (0)	1 (0.2)	0 (0)	1 (0.2)	0 (0)	1 (0.2)	0 (0)
**Rank**, ***n*** **(%)**					
Junior enlisted	69 (14.3)	65 (14.8)	4 (9.3)	63 (14.5)	6 (12.8)	64 (14.4)	5 (13.5)	63 (14.2)	6 (15.4)
NCOs	192 (39.8)	175 (39.8)	17 (39.5)	176 (40.4)	16 (34.0)	138 (41.0)	9 (24.3)	178 (40.1)	14 (35.9)
Staff NCOs	160 (33.1)	143 (32.5)	17 (39.5)	143 (32.8)	17 (36.2)	142 (31.8)	18 (48.7)	145 (32.7)	15 (38.5)
Officers	62 (12.8)	57 (13.0)	5 (11.6)	54 (12.4)	8 (17.0)	57 (12.8)	5 (13.5)	58 (13.1)	4 (10.3)
**History of combat deployment**, ***n*** **(%)**					
No	211 (43.7)	196 (44.6)	15 (34.9)	195 (44.7)	16 (34.0)	200 (44.8)	11 (29.7)	194 (43.7)	17 (43.6)
Yes	272 (56.3)	244 (55.5)	28 (65.1)	241 (55.3)	31 (66.0)	246 (55.2)	26 (70.3)	250 (56.3)	22 (56.4)
**# of combat deployment**,					
Mean (SD)	1.7 (2.2)	1.8 (2.3)	1.5 (1.5)	1.8 (2.3)	1.5 (1.5)	1.7 (2.2)	2.1 (1.9)	1.7 (2.2)	1.7 (2.0)
**Length of active duty (in years)**,					
Mean (SD)	11.5 (8.1)	11.4 (8.1)	12.2 (7.9)	11.5 (8.2)	11.6 (7.4)	11.4 (8.2)	12.7 (7.3)	11.6 (8.1)	11.0 (7.7)
**# of days in treatment**,					
Median (IQR)	109 (59–178)	**105 (57–175)**	**133 (74–−232)** ^**b**^	101.5 (57–176.5)	140 (107–196)	105 (57–176)	133 (91–224)	105 (58.5–175.2)	127 (76–210)
**# of months from injury to intake**					
Median (IQR)	20.9 (2.4–89.4)	19.0 (2.4–88.8)	56.3 (3.5–107.3)	18.8 (2.3–88.8)	48.6 (5.5–107.3)	**18.5 (2.3–87.3)**	**61.4 (7.5–107.3)** ^**b**^	19.9 (2.4–89.4)	34.5 (2.7–90.5)
**Pre-assessment outcome measures, mean (SD)**					
PHQ-8	11.4 (6.2)	**10.5 (5.7)**	**20.3 (3.2)** ^**b**^	**10.3 (5.5)**	**20.7 (3.3)** ^**b**^	**10.6 (5.7)**	**20.2 (3.8)** ^**b**^	**10.6 (5.8)**	**19.5 (3.8)** ^**b**^
PCL-5	30.9 (20.0)	**27.3 (16.9)**	**68.1 (7.4)** ^**b**^	**27.3 (17.1)**	**64.7 (11.7)** ^**b**^	**28.5 (18.3)**	**59.7 (17.1)** ^**b**^	**28.4 (18.3)**	**59.2 (16.3)** ^**b**^
NSI	35.3 (16.6)	**32.7 (14.3)**	**62.6 (13.0)** ^**b**^	**32.4 (14.2)**	**62.2 (12.4)** ^**b**^	**32.6 (13.8)**	**68.6 (9.4)** ^**b**^	**32.4 (13.7)**	**68.5 (9.3)** ^**b**^
**Post-assessment outcome measure, mean (SD)**					
PHQ-8	9.0 (6.7)	**8.3 (6.3)**	**16.3 (5.5)** ^**b**^	**8.2 (6.3)**	**16.6 (5.7)** ^**b**^	**32.6 (13.8)**	**68.6 (9.4)** ^**b**^	**8.5 (6.4)**	**14.6 (7.4)** ^**b**^
PCL-5	27.1 (21.5)	**24.1 (19.7)**	**57.2 (14.4)** ^**b**^	**24.5 (20.0)**	**51.3 (19.7)** ^**b**^	**10.6 (5.7)**	**20.2 (3.8)** ^**b**^	**25.2 (20.3)**	**48.1 (23.4)** ^**b**^
NSI	26.9 (17.9)	**25.0 (16.7)**	**47.2 (17.6)** ^**b**^	**24.8 (16.7)**	**46.5 (17.2)** ^**b**^	**25.5 (16.9)**	**44.1 (21.1)** ^**b**^	**25.2 (16.4)**	**46.4 (22.7)** ^**b**^

NMCSD, naval medical center, San Diego; SD, standard deviation; PHQ-8, patient health questionnaire, 8-items; PCL-5, post-traumatic stress disorder checklist, DSM-5; NSI, neurobehavioral symptom inventory; NCO, non-commissioned officers; IQR, interquartile range.

^a^High vs. low pre-treatment symptom score refers to having a score < 1.5 SD (low) or ≥1.5 SD (high).

^b^Significant *p*-value < 0.05 (bolded).

## 3. Results

Table 1 summarizes the characteristics of our study sample, overall and by the level of each symptom cluster prior to treatment. Overall, the mean age was 32.2 years (SD = 8.6), most were male (88.4%), Non-Hispanic White (65.6%) and married (67.7%). Approximately 35% completed an Associate's degree or higher. Most participants served in the Navy (46.6%) or Marine Corps (48.9%) and were either non-commissioned officers (NCOs, 39.8%) or staff NCOs (33.1%). The mean length of active duty was 11.5 years (SD = 8.1), and 56.3% of participants had been deployed in combat. The analysis identifying relative elevations (*z*-score ≥ 1.5) across the symptom clusters prior to treatment revealed the following: hyperarousal symptom cluster = 8.9%, dissociation/depression symptom cluster = 9.7%, cognitive /headache symptom cluster = 7.7%, neurological symptom = 8.1%. When demographic and military characteristics were evaluated by each symptom cluster, participants with high (vs. low) levels of the *hyperarousal* or *dissociation/depression* symptom cluster were more likely to be racial/ethnic minorities. The level of *cognitive/headache* symptom cluster did not differ by demographic or military characteristics, but it was associated with a longer length between injury and treatment initiation. Those with high (vs. low) levels of *neurological* symptom cluster were more likely to be non-Hispanic White. After excluding participants whose symptoms increased significantly with treatment, those with a high (vs. low) level of the *hyperarousal* or *dissociation/depression* symptom cluster were more likely to be racial/ethnic minorities and have a longer period of treatment. Those with a high (vs. low) level of the *cognitive/headache* symptom cluster had a longer period of treatment, and those with a high (vs. low) level of the *neurological* symptom cluster were more likely to be racial/ethnic minorities.

Table 2 provides the results evaluating the association between the level of each symptom cluster (in *z*-scores) and change in depressive, PTSD and neurobehavioral symptoms during treatment (i.e., the difference between the scores obtained during clinic intake and during discharge or the last follow-up). Overall, an *increase* in one standard deviation of *cognitive/headache* symptom cluster was significantly associated with a 6.7 unit *decrease* in neurobehavioral symptoms [CI = −12.6, −0.9]. The level of the *hyperarousal, dissociation/depression* and *neurological* symptom clusters were not associated with the rate of change in symptoms.

**Table 2:** Association between the level of symptom cluster (in *z*-score) prior to treatment and the rate of change in outcome (*n* = 483).

**Outcome measures**	**Symptom cluster prior to treatment** **Difference in mean change in outcome (95% confidence interval)**			
	**Hyperarousal**	**Dissociation/depression**	**Cognitive/headache**	**Neurological**
PHQ-8	1.5 (−1.2, 4.2)	1.3 (−0.5, 3.1)	−1.8 (−3.6, 0.1)	−0.6 (−2.4, 1.3)
PCL-5	0.01 (−8.3, 8.3)	−3.0 (−8.4, 2.4)	−5.1 (−10.4, 0.3)	−1.8 (−7.3, 3.6)
NSI	5.8 (−1.6, 13.3)	3.5 (−1.7, 8.7)	–**6.7 (**–**12.6**, –**0.9)**^**a**^	−3.4 (−9.5, 2.7)

PHQ-8, patient health questionnaire, 8-items (a measure of depressive symptoms); PCL-5, posttraumatic stress disorder checklist, DSM-5 (a measure of PTSD); NSI, neurobehavioral symptom inventory (a measure of neurobehavioral symptoms).

ANOVA, adjusted for the following by pre-treatment symptoms: hyperarousal: race, number of days in treatment, pre-treatment outcome level; dissociation/depression: race, pre-treatment outcome level; cognition/headache: number of months between injury and intake; and pre-treatment outcome level; neurological: race, pre-treatment outcome level.

^a^Significant *p*-value < 0.05 (bolded).

**Figure 2:**
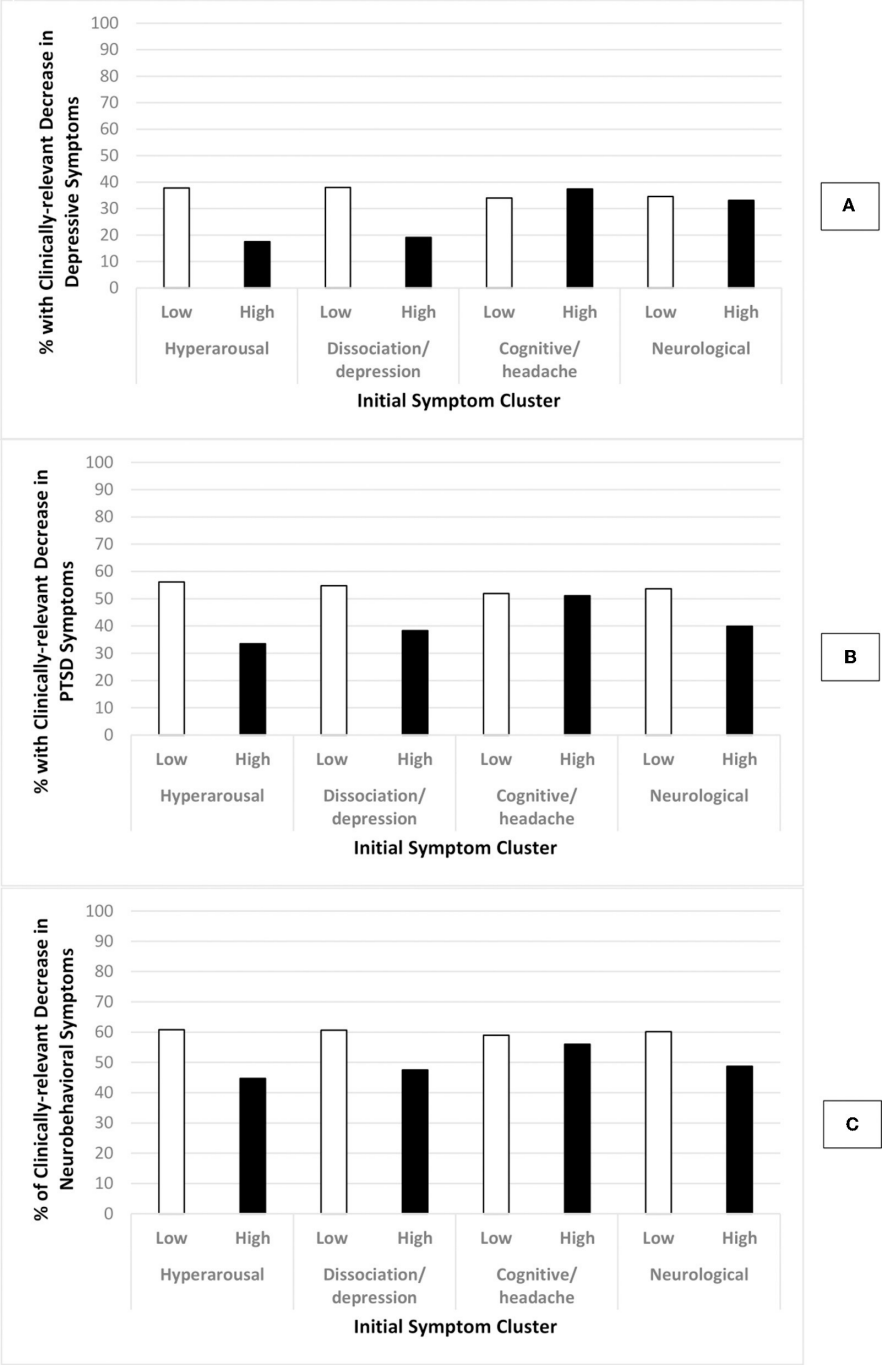
Percentage of participants with clinically-relevant DECREASE in outcome by level of initial symptoms. **Low initials symptoms**: total symptom cluster *z*-score < 1.5; **High initial symptoms**: total symptom cluster *z*-score ≥ 1.5; Graph **(A)**: change in depressive symptoms; Graph **(B)**: change in PTSD symptoms; Graph **(C)**: change in neurobehavioral symptoms; Clinically-relevant decrease in outcomes: PHQ-8 ≥ 5, PCL-5 ≥ 7, NSI ≥ 8.

Figure 2 illustrates the percentage of clinically-relevant decreases in self-reported depressive (Figure 2A), PTSD (Figure 2B) and neurobehavioral (Figure 2C) symptoms by level of hyperarousal, dissociation/depression, and cognitive/headache and neurological symptom clusters prior to treatment. The percentage with clinically-relevant *decreases* in depressive, PTSD and neurobehavioral symptoms were consistently lower for those with high (vs. low) levels of the *hyperarousal* and *dissociation/depression* symptom cluster prior to treatment. The differences between those with a high (vs. low) level of the *cognitive/headache* symptom cluster were negligible, while the percentage with clinically-relevant *decreases* in PTSD and neurobehavioral symptoms were lower for those with a high (vs. low) levels of the *neurological* symptom cluster.

**Figure 3:**
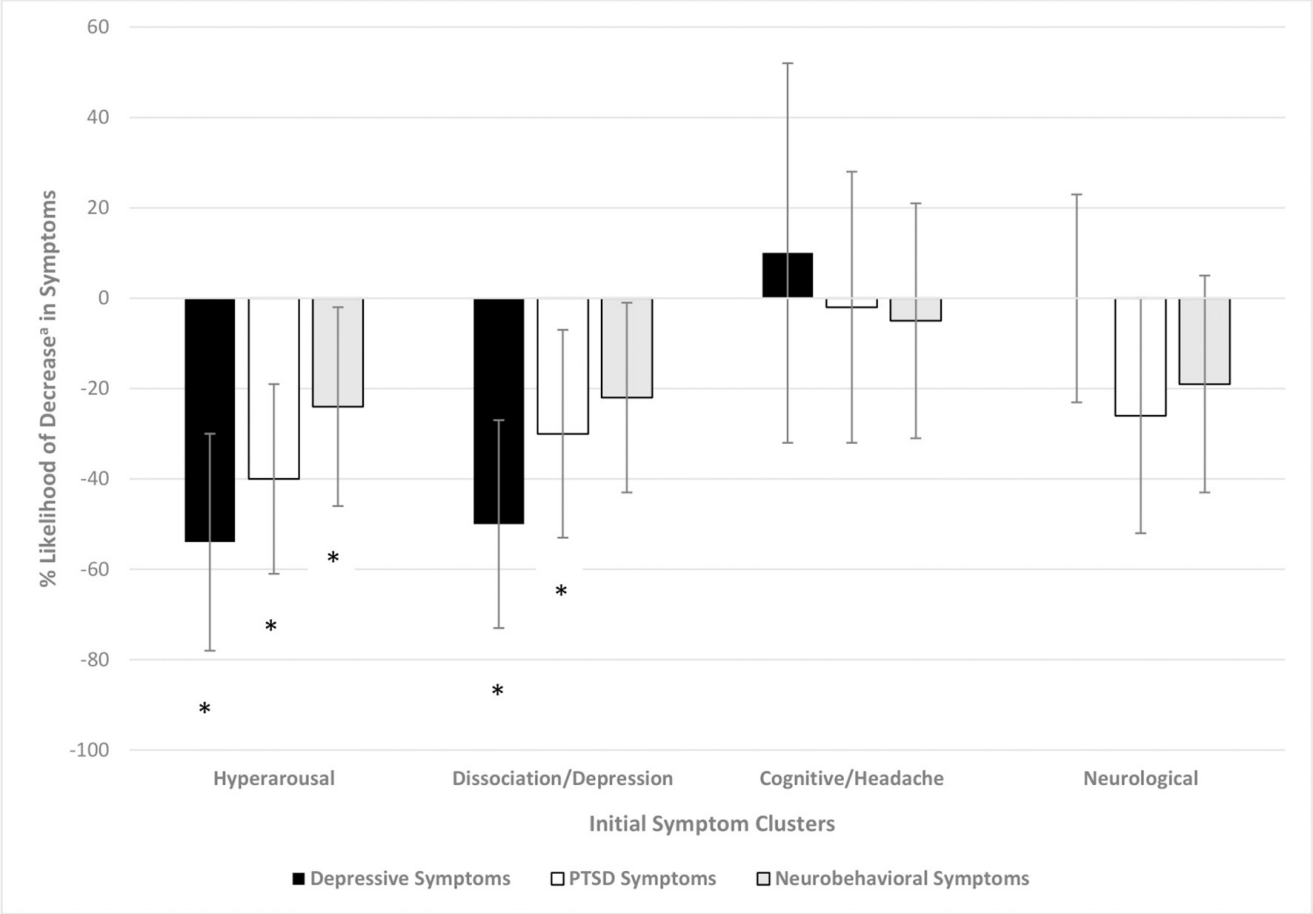
Percent likelihood of clinically-relevant decrease^a^ in outcome based on initial symptom cluster. Poisson regression with robust error variance, adjusted for the following by initial symptom cluster: hyperarousal: race, number of days in treatment, pre-treatment outcome level; dissociation/depression: race, number of days in treatment, pre-treatment outcome level; cognition/headache: number of days in treatment; and pre-treatment outcome level; neurological: pre-treatment outcome level. ^a^Clinically-relevant decrease of 5 points for PHQ-8, 7 points for PCL-5 and 8 points for NSI. *Significant *p*-value at the level < 0.05.

Statistical comparisons of differences presented in Figure 2 are provided in Figure 3. Participants with a high (vs. low) level on the *hyperarousal* symptom cluster were 54 (PR = 0.46, CI = 0.28, 0.76), 40 (PR = 0.60, CI = 0.43, 0.84), and 26% (PR = 0.74, CI = 0.55, 0.98) *less* likely to have a clinically-relevant *decrease* in depressive, PTSD and neurobehavioral symptoms, respectively. Those with high levels of the *dissociation/depression* symptom cluster were 50 (PR = 0.50, CI = 0.32, 0.78) and 30% (PR = 0.70, CI = 0.51, 0.96) *less* likely to have a clinically-relevant *decrease* in depressive and PTSD symptoms, respectively. The levels on the *cognitive/headache* or *neurological* symptom cluster were not associated with decreases in depressive, PTSD or neurobehavioral symptoms.

Sensitivity analyses between patients with vs. without discharge outcome measures found similar trends for *hyperarousal, dissociation/depression* and *cognitive/headache* symptom clusters, but not for *neurological* symptom clusters (see Supplementary Figure 1). Specifically, among those with discharge data, participants with high (vs. low) level of *hyperarousal* and *dissociation/depression* symptom clusters at intake were less likely to decrease in mental health symptoms, and no significance was found with *cognitive/headache* symptom cluster. Although the same trend was found for *neurological* symptom clusters whereby those with high vs. low levels of *neurological* symptom clusters were less likely to decrease in PTSD and neurobehavioral symptoms (similar to the results from the pooled data), it was statistically significant among those with discharge data.

## 4. Discussion

The findings of this study demonstrated that pre-treatment symptom severity can serve as a valuable prognostic tool on treatment outcomes among active duty service members in an interdisciplinary TBI rehabilitation program. Specifically, patients who began treatment with relatively high levels of *hyperarousal* and/or *dissociation/depressive* symptoms were *less* likely to benefit from interdisciplinary TBI treatment; they were less likely to have improvements in their mood (both depression and post-traumatic stress) and less likely to have reductions in overall neurobehavioral symptoms. More specifically, when considering clinically-relevant decreases in symptoms, elevated scores on the *hyperarousal* and/or *dissociation/depressive* symptom clusters were less likely to have a clinically-relevant improvement in depressive, PTSD and neurobehavioral symptoms following treatment. Patients who began treatment with relatively high levels of *cognitive* complaints and *headaches* were more likely to have a positive response to treatment with greater reductions in overall neurobehavioral symptom burden.

The subset of service members who present with elevated mental health symptoms may have psychiatric needs that extends beyond the resources of a TBI-oriented treatment program. Those with severe symptoms such as PTSD may be receiving their mental health needs through the Mental Health Department within the Department of Defense that is outside of TBI care. Though all participants in this study were enrolled in interdisciplinary treatment programs that offered some aspect of mental health-oriented services (e.g., individual psychotherapy, mindfulness training), the percentage who were referred for these services or engaged in treatment were unknown. Generally speaking, access to mental health services may not be widely available for military personnel with a history of TBI (38). It is recognized that stigma against mental health conditions may hinder treatment seeking or utilization of available treatments being offered (39–41). As such, this may limit the potential benefits of an interdisciplinary TBI rehabilitation to effectively improve mental health symptoms (20); however, given that the overall sample did have improved psychiatric symptoms after treatment, there may be something particular about the patients who present with relatively high symptoms. These psychiatric symptoms may be of a severity that extends beyond the resources of a TBI-oriented treatment program.

Identifying characteristics associated with poor recovery, such as those related to mental health in the present study, may enable early identification and intervention, leading to improved outcomes *via* personalized medicine. Our findings may suggest that treatment within a program for TBI (including concussion) may be bolstered by stronger collaborations with mental health treatments either before or in congruence with TBI rehabilitation. In addition to improving the presence of mental health providers and treatment in the management of a patient's TBI recovery, education that reduces stigma against mental health illness may also improve acceptance and compliance with mental health treatment.

### 4.1. Limitations

A few limitations need mentioning. First, the variables analyzed in this study were based on self-report, which is inherently prone to biases such as issues with recall and reporting. However, these assessments have been well-validated and allow for evaluation of several factors without overburdening study participants. Post-treatment data were also obtained from either the last follow-up available during treatment or from measures administered during the discharge process. Such differences may overestimate the increase in symptoms as it is possible that some patients with no discharge data actually improved after their last measurement time point and self-discharged without completing treatment and outcome post-treatment measures. However, this was not the case when we conducted sensitivity analyses comparing results using the entire sample to those with only discharge data. Among those with discharge data, we continued to see less improvement among those with *hyperarousal* and *dissociation/depression* symptom clusters. Nonetheless, we accounted for the length of treatment in models where applicable to mitigate the potential impact of this issue. Additionally, it is important to note that mental health symptoms at clinical intake and during treatment may not have been specifically derived from the diagnosed concussion (i.e., patients could have had premorbid depression or PTSD prior to their concussion), and no data was available for the level of depressive and PTSD symptoms prior to clinical intake for the treatment of concussion. It is, thus, unknown whether participants with prior diagnoses of major depression and/or PTSD may have been those reporting high levels of *hyperarousal* and *dissociation/depression* symptom clusters. However, as our study focus was on whether such high levels of symptom clusters, regardless of their origins (i.e., whether present prior to concussion or triggered by such event), impacted mental health symptoms during treatment, our findings would still support the need to address mental health symptomatology during treatment for TBI. We also did not have access to utilization records to determine the amount of engagement with mental health-oriented services, particularly outside of the TBI program, or the type or frequency of treatment utilized. This information can further provide insight into the strength of mental health services in addressing mental health needs of TBI patients. Symptom clusters were also originally determined using PCL-C by Bailie et al.; however, our study utilized items from PCL-5 as this was the version available for this study. Although items were similar in verbiage, a future study which utilizes PCL-C items to determine categories of symptom clusters is needed to validate the findings of this study. A cut-off for clinically-relevant decrease in depressive symptoms was also based on a study utilizing PHQ-9 and not PHQ-8; however, this would only underestimate our findings as there would be 3 less potential points to accrue using PHQ-8 compared to PHQ-9. Similarly, the cut-off scores for clinically-relevant decrease in PTSD symptoms were determined using PCL-C by Belanger et al. (34); however, these cut-offs were applied to scores obtained in this study from the PCL-5. Although these instruments were different, they have been shown to have “substantial to excellent agreement” when sum scores were compared (42). The summary score also ranges from 0 to 80 for PCL-5, compared to 17–85 for PCL-C. As such, the use of PCL-5 would likely underestimate the findings. Nonetheless, future studies are needed to directly validate the cut-off for a clinically-relevant decrease in PTSD using PCL-5. Although we were able to adjust for multiple covariates, residual confounding may also still exist as these measures may not have fully adjusted out their effects. Additionally, as the symptom clusters were developed using the PCL-5 and NSI, these were not exhaustive of other existing conditions (e.g., pain, insomnia) prior to treatment that may impact mental health and neurobehavioral symptom recovery. This study also evaluated outcomes individually, although we acknowledge that outcomes may exist simultaneously (e.g., clinically-relevant decreases in depressive, PTSD and neurobehavioral symptoms) and synergistically impact the results. For example, those with clinically-relevant decreases in one psychiatric symptom would likely have a clinically-relevant decrease in the other (as well as other post-concussive symptoms), and such improvement would likely be greater in magnitude compared to those with improvement in only one outcome. As maintaining ample sample size for our analyses would be problematic if we considered clinically-relevant decreases in depressive, PTSD and neurobehavioral symptoms concurrently, we were not able to evaluate these outcomes simultaneously in the current analyses. Studies with larger sample sizes are warranted to appropriately account for these concurrent outcomes. Lastly, as our study sample consisted predominantly of Non-Hispanic White male military service members, generalizability of findings will be limited to similar populations. Additional large studies with differing demographic characteristics are warranted to potentially validate our findings and expand generalizability to other populations.

## 5. Conclusions

The findings from this study suggest that, within the context of a military concussion rehabilitation program, higher levels of *hyperarousal* and *dissociation/depression* at clinic intake are associated with diminished improvements in depressive, PTSD and neurobehavioral symptoms with treatment. Potentially, increased emphasis on individualized treatment of these mental health symptoms early in the treatment process as part of their TBI care, or prior to such care, may help decrease the burden of mental health issues among service members receiving treatment in TBI rehabilitation settings.

## Data availability statement

The datasets presented in this article are not readily available because of ethical and privacy restrictions. Information and the policies regarding limitations on sharing DoD/DHA data publicly, without an approved Data Sharing Agreement Application (DSAA), can be found at the following website (https://www.health.mil/Military-Health-Topics/Privacy-and-Civil-Liberties/Submit-a-Data-Sharing-Application). The specific DoD Directive (DoDD) that speaks to why we cannot simply share data, even a minimal, de-identified dataset, is DoDD 5400.11 (please refer to additional documentation titled DoDD-5400.11). In order to access DoD/DHA data, a DSAA must be submitted to the Privacy Office. The appropriate point of contact to initiate the DSAA process can be reached at DHA.DataSharing@health.mil. This DSAA would be between TBICoE and the intended recipient of the data and would need to be requested by the recipient and signatures obtained from all party's authorities. The DSAA would outline the intended use and retention of the data, which will be reviewed by the Privacy Board (estimated review time is between 3 and 6 months depending on the type of request). A determination will then be made by the Privacy Board based on whether the intended use of the data by the recipient meets the standards of the DoD Privacy Program. Approval of DSAA is subject to Privacy Board review. For further information, questions can be submitted to the Privacy Office at DHA.DataSharing@health.mil. Requests to access the datasets should be directed to the DoD Privacy Office, DHA.DataSharing@health.mil.

## Ethics statement

The studies involving human participants were reviewed and approved by Naval Medical Center San Diego IRB Committee. Written informed consent for participation was not required for this study in accordance with the national legislation and the institutional requirements.

## Author contributions

RR-B: conceptualization (equal), methodology (lead), software (lead), validation (lead), visualization (lead), writing—original draft (lead), and writing—review and editing (equal). LH, ME, and IB: project administration (equal) and writing—review and editing (equal). LB: writing—original draft (supporting) and writing—review and editing (equal). BI: writing—review and editing (equal). KS: project administration (equal) and writing—review and editing (equal). CM: data curation (lead). JB: conceptualization (equal), project administration (equal), and writing—review and editing (equal). All authors contributed to the article and approved the submitted version.
