# Supplementary material for: Presenting symptoms as prognostic measures of mental health recovery among service members with concussion
Source: Front Neurol. 2023 Jan 13;13:1070676. doi: 10.3389/fneur.2022.1070676 (PMC9880328; doi:10.3389/fneur.2022.1070676)
Supplement: Supplementary file 1 [file Table_1.docx]

**Supplementary Table 1. Adaptation of NSI and PCL-5 question item classification into categories/clusters of initial symptoms (hyperarousal, dissociation/depression, cognitive/headache, and neurological) as determined by Bailie et al.**

| **Measure** | **Item** | **Question Item** | **Categories of Initial Symptoms** | | | |
| --- | --- | --- | --- | --- | --- | --- |
|  |  |  | **Hyperarousal** | **Dissociation/ Depression** | **Cognitive Dysfunction/ Headache** | **Neurological** |
| PCL-5 | 2 | Disturbing dreams of stressful events | X |  |  |  |
| PCL-5 | 1 | Disturbing memories of stressful event | X |  |  |  |
| PCL-5 | 3 | Feeling stressful events were happening again | X |  |  |  |
| PCL-5 | 17 | Feeling jumpy or easily startled | X |  |  |  |
| PCL-5 | 16 | Being “super alert” or on guard | X |  |  |  |
| PCL-5 | 4 | Feeling upset when reminded of stressful event | X |  |  |  |
| PCL-5 | 5 | Having physical reactions | X |  |  |  |
| PCL-5 | 6 | Avoid thinking or talking about stressful event | X |  |  |  |
| PCL-5 | 20 | Trouble falling or staying asleep | X |  |  |  |
| PCL-5 | 7 | Avoid situations because they remind of stressful event | X |  |  |  |
| NSI | 18 | Difficulty falling or staying asleep | X |  |  |  |
| PCL-5 | 13 | Feeling distant from others |  | X |  |  |
| PCL-5 | 14 | Feeling emotionally numb |  | X |  |  |
| PCL-5 | 12 | Loss of interest in things you used to enjoy |  | X |  |  |
| NSI | 20 | Feeling depressed or sad |  | X |  |  |
| PCL-5 | 9 | Feeling your future will be cut-short |  | X |  |  |
| PCL-5 | 15 | Feeling irritable or having angry outbursts |  | X |  |  |
| NSI | 22 | Poor frustration tolerance |  | X |  |  |
| NSI | 21 | Irritability, easily annoyed |  | X |  |  |
| NSI | 19 | Anxious or tense |  | X |  |  |
| PCL-5 | 8 | Trouble remembering important parts of stressful event |  | X |  |  |
| NSI | 14 | Forgetfulness |  |  | X |  |
| NSI | 4 | Headaches |  |  | X |  |
| NSI | 13 | Poor concentration or easily distracted |  |  | X |  |
| NSI | 16 | Slowed thinking/organization/can’t finish |  |  | X |  |
| NSI | 7 | Sensitivity to light |  |  | X |  |
| PCL-5 | 19 | Difficulty concentrating |  |  | X |  |
| NSI | 15 | Difficulty making decisions |  |  | X |  |
| NSI | 9 | Sensitivity to noise |  |  | X |  |
| NSI | 8 | Hearing difficulty |  |  | X |  |
| NSI | 11 | Change in taste and/or smell |  |  |  | X |
| NSI | 10 | Numbness or tingling in parts of body |  |  |  | X |
| NSI | 2 | Loss of balance |  |  |  | X |
| NSI | 6 | Vision problems |  |  |  | X |
| NSI | 5 | Nausea |  |  |  | X |
| NSI | 1 | Feeling dizzy |  |  |  | X |
| NSI | 12 | Loss or increase in appetite |  |  |  | X |
| NSI | 3 | Poor coordination |  |  |  | X |
| NSI | 17 | Fatigue |  |  |  | X |

Abbreviations: PCL-5, PTSD Checklist, DSM-5; NSI, Neurobehavioral Symptom Inventory

Adapted from Bailie et al. Profile analysis of neurobehavioral and psychiatric symptoms following combat-related mild traumatic brain injury: Identification of subtypes. *J Head Trauma Rehabil*. 2016;31(1):2-12.

NOTE: Bailie et al utilized the PCL-C which is the Civilian version of the PCL-5. The PCL-5 items listed in the table above is the corresponding item with PCL-C.
