# Supplementary material for: Presenting symptoms as prognostic measures of mental health recovery among service members with concussion
Source: Front Neurol. 2023 Jan 13;13:1070676. doi: 10.3389/fneur.2022.1070676 (PMC9880328; doi:10.3389/fneur.2022.1070676)
Supplement: Supplementary file 2 [file Image_1.pdf]

Supplemental Figure 1. Percent likelihood of clinically-relevant decrease[a] in outcome based on initial symptom cluster – Limited to sample with discharge data.

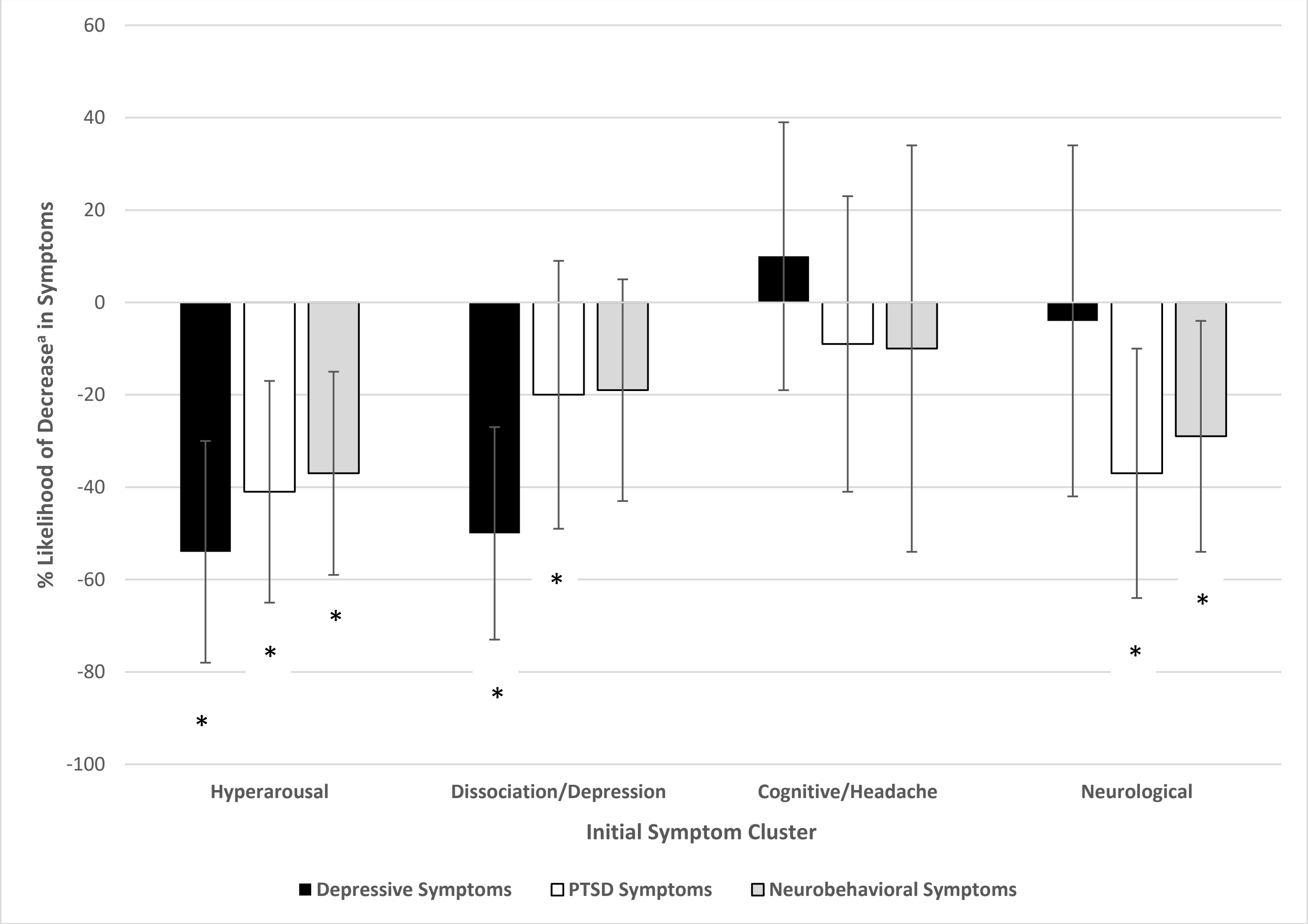

NOTE: Poisson regression with robust error variance, adjusted for the following by initial symptoms: Hyperarousal: race, number of days in treatment, pre-treatment outcome level; Dissociation/depression: race, number of days in treatment, pre-treatment outcome

level; Cognition/headache: number of days in treatment, pre-treatment outcome level; Neurological: race, pre-treatment outcome level

[a] Clinically-relevant decrease of 5 points for PHQ-8, 7 points for PCL-5 and 8 points for NSI.

*Significant p-value at the level <0.05
